# Conflicting demands for clinical practice (CP) and continuous quality improvement (CQI) in emergency departments (EDs)

**DOI:** 10.1108/JHOM-05-2025-0226

**Published:** 2025-12-26

**Authors:** Kristina Mitreska, Milé Terziovski

**Affiliations:** Department Business Technology and Entrepreneurship, Swinburne University of Technology, Hawthorn, Australia; Swinburne University of Technology, Hawthorn, Australia; Western Health, Melbourne, Australia

**Keywords:** Emergency departments, Healthcare, Tension, Paradox, Hybrid leadership, Continuous improvement, CQI, Patient care, Leadership, Performance outcomes

## Abstract

**Purpose:**

Emergency department (ED) physicians are expected to effectively implement continuous quality improvement (CQI) practices in EDs while providing clinical service at the same time. This causes tension that often results in unsuccessful CQI implementation in EDs, leading to increased costs and disgruntled patients and staff. The purpose of our study is to explore the underlying factors that contribute to the paradox faced by ED physicians in balancing their clinical duties and CQI expectancies to improve process efficiencies in Australian EDs.

**Design/methodology/approach:**

The methodology is based on convergent interviews of 16 physicians of Australian EDs. To share their perceived challenges in dealing with clinical and CQI practice of the paradox and how ED physicians dealt with the challenge.

**Findings:**

The qualitative data reveal ED physicians’ appetite to implement CQI in their EDs. The results also unveiled significant gaps in physicians’ CQI knowledge and the need for the allocation of more non-clinical time to ED physicians to undertake CQI activities to build a culture of continuous improvement. This would minimise “tension” and improve perceived ED performance outcomes.

**Practical implications:**

Results would equip ED physicians with a greater understanding of the complexities and tensions surrounding EDs when adopting CQI strategies, while providing clinical care to meet community expectations and to reduce costs.

**Originality/value:**

The paper is original in its methodology and research findings. The study provides a theoretical framework as part of the methods of the interview to assist ED physicians to address the tension between clinical and CQI practice.

## 1. Introduction

The Australasian College for Emergency Medicine Position Statement (ACEM, 2021b) identified the presence of “*significant acute health system challenges*” that adversely affect the operational capabilities of emergency department (EDs), hindering their ability to function efficiently and effectively (p. 4). The ACEM is a not-for-profit establishment that provides emergency physicians training, offers ongoing education and enhances professional standards in ED in Australia and New Zealand. ACEM identifies tension between health policy and resource allocation causing overcrowding and insufficient beds while patients wait for hospital admissions (ACEM, 2021a).

The tension relates to professional independence, workforce resistance and self-surveillance, shaped by political and organisational contexts (Ferlie *et al.*, 2012). On the other hand, ED physicians are highly committed professionals, who provide their patients with the highest quality care delivered within the constraints of available resources. Furthermore, the tension has a detrimental impact on the quality and safety of patient care in EDs, causing dissatisfaction and reduced staff morale (ACEM, 2021a). Thus, ACEM recommends a systems approach and implementation of transformational change *“with the identification of system-wide clinical process redesign solutions tailored to local needs”* (ACEM, 2021b, p. 5). Therefore, the purpose of our study is to explore the underlying factors that contribute to the paradox faced by ED physicians in their challenge to balance clinical duties with an approach to improve process efficiencies in Australian EDs.

A “paradox” entails an ongoing commitment to deal with various conflicting demands, or tensions. Smith and Lewis (2011, p. 386) define a paradox as *“contradictory yet interrelated elements that exist simultaneously and persist over time”*. According to the authors, tensions operate between and within the fundamental organisation’s activities of “*learning, belonging, organising, and performing”* (Smith and Lewis, 2011), all relevant to the successful implementation of CQI.

Consequent to the above, ED physicians’ ability to effectively implement CQI practices in EDs while providing clinical service creates a paradox that often leads to unsuccessful CQI implementation in EDs, resulting in increased costs and disgruntled patients. Alexander *et al.* (2006, p. 1008) articulated the critical importance for ED physicians to address the paradox “*as a mechanism to provide high-quality care while containing health care cost.”*

Therefore, the aim of this study is to explore the factors contributing to the paradox faced by ED physicians in balancing their clinical duties and CQI expectancies to improve process efficiencies in Australian EDs, which would ultimately minimise “tension” and improve perceived ED performance outcomes.

Accordingly, this study addresses the following question:

What are the critical factors that contribute to the paradox faced by ED physicians and how do the conflicting demands for clinical practices (CP) and CQI the physicians manage simultaneously?

## 2. Theoretical foundation

The theoretical positioning is based on Eisenhardt (1989) seminal article that outlines steps for building theory from case studies research. The following theoretical framework was developed to compliment the interview process to gain a deeper understanding on how and where the tension arises and how physicians should manage CQI and CP (Jepsen and Rodwell, 2008).

### 2.1 The need of CQI practice in Australian EDs

The mismatch and unsteadiness between health policy and resource allocation with current ED healthcare demand increases the risk of errors, overall health system costs and near misses or an event that might result in harm (ACEM, 2021a, b). This also causes delays and increased risks in the provision of care, which often results in decreased patient satisfaction, welfare and staff morale (ACEM, 2021a, b).

The current ED climate of healthcare cost increases and high patient demand urges Australian ED directors to redistribute resources to more cost-effective and efficient CP strategies, to provide better quality of patient care (Day *et al*., 2016).

CQI practices challenge traditional thinking, sparking creativity in problem-solving and assisting in realigning operational performance (Berghout *et al.*, 2017). CQI practices help to facilitate open communication and more cost-effective CP that enables improvement of operational performance parameters that are aligned with patient care (Imran *et al.*, 2021; Orlando, 2020).

### 2.2 Concepts and definitions of CQI

CQI research shows that creating an environment that supports CI practice is beneficial to an organisation’s culture and is one of the main predictors of innovation and operational performance (García-Buades *et al.*, 2015). Such research and practice have acknowledged the presence of various improvement methodologies, such as Lean Management, Business Process Re-engineering (BPR), Total Quality Management (TQM), Six Sigma and others. While all these CI concepts have been found to be complementary and share common goals and origins, Lean Management has been recognised as one of the most popular business improvement strategies used internationally. Research has found that CI methodologies help organisations to integrate, collaborate and continuously improve and sustain performance outcomes. Thus, this research has focused mainly on the investigation and understanding of the CQI concept and its implementation in the ED sector.

Lean Management implementation has been presented in several research studies that investigate its implementation challenges (Dickson *et al.*, 2009, p. 1; Jones *et al.*, 2006 cited in Lindsay *et al.*, 2020, p. 3). This study focused on the application of CQI management, specifically in ED settings. Accordingly, this paper has embraced the systematic organisational focus of CQI in EDs, defining it as: “*a long-term management strategy to develop an internal culture of ongoing organisational learning process used to create the most value for customers (patients), while all contestants in the chain of value of the provided service actively participate in identifying and reducing non-value activities (waste) and emphasise the culture of empowerment, responsibility, teamwork and knowledge building across the whole patient flow”.*

However, Dickson *et al.’s* (2009, p. 6) research of Lean implementation cases across multiple EDs concludes that “*Lean is rather a tool that may or may not succeed, according to the efforts surrounding its use*”. The authors further suggested that Lean’s unpredictable results could point towards a “context + mechanism = outcome” in which “context” implies to an ED and “mechanism” relates to Lean (Dickson *et al.*, 2009). However, the authors fall short to explain why Lean produces unpredictable results in EDs. It is reasonable to assume at this stage this could be due to the conflicting demands for CP and CQI.

### 2.3 ED physicians’ resistance when adopting CQI

Despite the recognised need for enhancing efficiencies in EDs, physicians face challenges in effectively implementing CQI practices (Mannion and Davies, 2018; Sartirana *et al.*, 2019). Evidence from the literature documents unsuccessful CQI implementation in EDs, underpinned by resistance by physicians to adopt change and to embrace full engagement in CQI activities. Physicians perceive the paradoxical roles as incompatible (Mannion and Davies, 2018; Sartirana *et al.*, 2019).

Research by Sartirana *et al. (*2019) has attributed these challenges to a different mindset that physicians have developed because of their long-established CP (Sartirana *et al.*, 2019). The physician’s mindset often leads to conflicting priorities and competing logic when undertaking required clinical and managerial duties. Other potential causes reported in the literature are typically due to role ambiguity, insufficient training and lack of time allocated for CQI (Berghout *et al.*, 2017; Spurgeon *et al.*, 2015). Furthermore, the literature suggests that the professional responsibilities of today’s clinicians are complex and often compromised, creating a “tension” between two distinct mindsets, both in principle and practice (Kippist and Fitzgerald, 2009). Ferlie *et al.* (2012) explain the continuous improvement (CI) implementation tension as *“inappropriately imported models of private sector management”* lacking clarity with fragmented understanding and perplexed implementation. Lameijer *et al.* (2023) reinforce Ferlie’s view by arguing that CQI *“takes little account of the distinctive character of public sector organisations”* (p. 1302), which has largely led to implementation failure of CQI in health care.

### 2.4 Managing the paradox

We have explored Smith and Lewis’s (2011) organisational “paradox” model to examine the underlying factors that contribute to the paradox faced among healthcare professionals in balancing their clinical duties and CQI obligations. However, these efforts create tensions through the dynamic process of employing new concepts, between either radical and incremental improvement or random and constant change (O’Reilly and Tushman, 2008). This means that when ED physicians need to undertake CQI management roles, they must manage competencies they may not necessarily possess, as these have not been part of the usual medical school curriculum.

Managing paradoxes raises tensions, such as *“collaboration-control, individual-collective or collaboration-competition, flexibility-efficiency, exploration-exploitation and profit-social responsibility”* (Smith and Lewis, 2011, p. 381). How physicians respond to these tensions may be a determining factor for the organisation’s success. For example, CQI practice, underpinned by standardisation, simplicity and efficiency, while fostering flexibility, CI and agility, poses a unique challenge and causes potential conflict within the healthcare system, where physicians often seek control and autonomy. However, tensions arise among physicians due to performance expectations as they move into hospital management roles, including CQI. The tension reflects the complicated balance required in the physician’s pursuit of improvement of patient care delivery.

Literature further explore complexity as a driver of tension in EDs, which requires staff to possess both clinical and management competencies to be effective (Loh, 2015). For example, the transition to a management role often causes internal tensions between the physician’s perceived identity as managers and medical practitioners, whose number one priority is to care for their patients (Loh, 2015). In summary, the research has revealed that the effort to substitute professional bureaucracies in hospitals with operational business models has been ineffective (Loh, 2015). Consequently, the investigation has identified the need to explore the underlying factors that contribute to the paradox faced by ED physicians in balancing their clinical duties and CQI expectancies to improve process efficiencies in Australian EDs. In particular, this research has identified the need for exploring how recurring physicians’ leadership responses and competencies to paradoxical CQI ED change tensions enable CQI success and sustainability in the future (Smith and Lewis, 2011).

Finally, while there is an abundance of literature that provides prescriptive and complex solution models on how to manage CQI, the literature and empirical evidence lack a convincing explanation as to why organisations, including the health sector, have failed or struggled to implement and achieve intended performance, service delivery and capability uplift when implementing CQI practices (Dickson *et al.*, 2009). Thus, little has been published about healthcare organisations that have failed to achieve intended behavioural changes and openly discuss and elaborate reasons for their failure (Dickson *et al.*, 2009). Subsequently, this study addresses this knowledge gap.

## 3. Methodology

The qualitative methodology in this study aims to explore how CQI and CP have been managed by ED physicians within established Australian ED organisational structures, policies and processes, as part of their dual ED roles and responsibilities. This overall qualitative data component enabled the identification of key issues across all EDs, believed to have a significant influence on physicians’ performance and decision-making.

The methodology is based on Jepsen and Rodwell’s (2008) convergent diagnostic interviewing technique, involving interview design, participant selection and undertaking of convergent interview process, interview round analysis and final issue assessment. This qualitative methodology approach of conducting convergent interviews helped to identify gaps and contradictions between obtained qualitative data findings and initial literature research findings. The theory building process included interviews, supported with observations and archival sources, such as filed notes and probing questions (Eisenhardt, 1989). The theoretical model developed earlier enabled the researchers to apply the convergent interview process within a specified framework to gain a deeper understanding of the tension that arises within EDs due to the conflicting ED physician role priorities.

### 3.1 Data collection process: convergent interview method

To follow up on emerging themes from the literature review and assess the ED CQI practical implementation, face-to-face semi-structured convergent interviews were conducted with selected ED participants, from the ACEM database. The data collection process commenced once all the arrangements and approvals from an appropriate stakeholder were obtained. This includes the ACEM granted *“Letter of Endorsement”* to approve and support this research project officially and allowing Australian ED physicians to participate. In addition, the Swinburne University Human Research Ethics Committee (SUHREC) granted the approved ethical application for conducting all necessary data collection for this study.

### 3.2 Participant selection and recruitment

Participants were selected based on a convergent interview process which commenced with the distribution of an initial *“Information statement”* by an ACEM representative to all current ACEM members, outlining the interview process prior to the commencement of each interview. However, to protect privacy and confidentiality, the ACEM was not further advised of actual participant response to the initial invitation for participation and participant involvement in the data collection process. This mechanism of voluntary self-selection allowed potential participants to decide whether they wished to participate and further engage with the researcher by arranging a time to undertake the interview. They were further assessed by the researcher for selection according to their involvement in CQI activity within their EDs and ensuring both rural and metro representation.

Access to interview participants was obtained by formally arranged meetings with each interviewee who agreed and responded to the initial ACEM invitation for participation, and confidentiality of information collected was guaranteed. The interviews were arranged at a mutually agreed place and time.

For those interested in taking part, a “*Consent Information Statement*” was shared as part of this recruitment process, which gave potential participants information about the study. At the same time enabled them to choose to decline the invitation to participate via the participant interview recruitment email and consent information statement before the commencement of the actual interview.

### 3.3 Undertaking of the convergent interview process

Four convergent, individual open-ended interview questions and 19 sub-questions were developed to explore physicians’ CQI journey experience. The first convergent interview questions were open-ended, and their clarity was presented in a way that allowed a building of rapport with interview participants at interview initiation, followed by discussions focusing on CQI and change practice more generally. As the interview progressed, questions were more adapted and specific to participants’ CQI perceptions, focusing on direct experiences with CQI activities and constructs identified through building the conceptual and theoretical framework process. Each interview mainly took an hour to reach the level of detail needed to identify key issues. The interview discussion was concluded by summarising key issues raised by the respondent.

Post-interview, a list of main topics identified by respondents was obtained and summarised, sometimes including up to 10 issues recognised during the discussion. This structured approach enabled the identification of key issues perceived to have a significant impact on EDs operational performance (Jepsen and Rodwell, 2008). According to Jepsen and Rodwell (2008) research, convergent interviewing is an effective, robust and organised method for collecting a high volume of qualitative data from a diverse population and identifying key issues for a selected research topic.

The interviews were conducted both in person and via the Zoom platform, with each session lasting approximately one hour. Most interviews (13) were undertaken using the Zoom platform that was deemed as appropriate technique for interviewing participants in other states and remote rural areas. This approach helped to overcome any inconvenience due to the time and geographical distance limitations of most participants in this research study. Archibald *et al.* (2019) work support the view of Zoom’s “*interview experience as highly satisfactory and … rated Zoom above alternative interviewing mediums such as face-to-face, telephone, and other videoconferencing services, …*” (p. 1). The authors’ favourability on Zoom viability have been attributed to “*its relative ease of use, cost-effectiveness, data management features, and security options*.” (Archibald *et al.*, 2019, p. 1).

The sample size was selected from a population of Australian ED physicians. A total of 23 physicians agreed to participate in the interview process. Both metro and rural representatives were included. However, the number of interviews conducted was determined by reaching saturation point, with 16 participants interviewed. Additional insights did not emerge from subsequent interviews, leading to the conclusion of the convergent interview process.

This data collection technique *“was designed to develop theories of success and failure,”* as recommended by Eisenhardt (1989, p. 537). Thus, the interview sample covered only six of the participants who had CQI expertise, and 10 participants who had no prior knowledge or CQI experience. It also included participants from various Australian states, with the majority (94% or *n* = 15) of participants within the 40–60-years age range. The sampling plan provided insight into the level of ED physicians’ *“tension”* experienced between CQI and CP, which affected their perceived ED performance outcomes. The conclusive profile of 16 ED physicians is presented in Table 1.

**Table 1:** Profile of convergent interview participants

Respondent	Round	ED physician level	Tenure	Age group	Sex	State	Rural/Metro	CI experience	FT/PT
EMP1	1	Director/Senior Lecturer	18	40–50	M	NSW	Metro	No, self-thought	FT
EMP2		Director	17	50–60	M	NSW	Metro	Yes	FT
EMP3		Director	20	40–50	M	VIC	Metro	No, self-thought	FT
EMP4		Emergency Physician	21	50–60	F	NSW	Metro	No	PT
EMP5	2	Director	21	50–60	F	VIC	Metro	Yes	FT
EMP6		Emergency Physician	12	40–50	M	QLD	Metro	Yes	FT
EMP7		ED Consultant	12	50–60	M	WA	Metro	No	FT
EMP8		Director	18	50–60	M	NT	Metro	Yes	FT
EMP9	3	Director	12	40–50	M	QLD	Metro	No	FT
EMP10		ED Consultant	14	50–60	F	WA	Metro	No	FT
EMP11		Director	13	40–50	M	VIC	Metro	Yes	FT
EMP12		Chief of Critical Care/Associate Professor	25	50–60	M	VIC	Metro	No, self-thought	FT
EMP13	4	ED Consultant	8	30–40	F	NSW	Rural	No	FT
EMP14		Emergency Physician	10	40–50	M	NT	Rural	No	FT/Contract
EMP15		Director	3	40–50	F	QLD	Rural	No	PT
EMP16		Director	23	40–50	M	VIC	Rural	Yes	FT

**Source(s):** Authors’ own work

Fourteen out of the sixteen physicians interviewed were employed on a full-time basis. One participant was on a contract, and one participant was continuing part-time. The average work experience of the respondent was 15 years, ranging from 3 years to 25 years. Eighty-eight per cent (*n* = 14) of the participants had 10 or more years of experience within Australian hospitals as ED physicians. Sixty-three per cent (*n* = 10) held a role as ED director. Their years of experience were not found to be related to the position they held. There was no response from the junior ED physicians to the convergent interview invitation. Therefore, it is reasonable to assume that junior doctors usually are focussed on patient care as part of their training and did not engage in CQI activities. The process of qualitative data collection was finalised within one month. The following step to convergent interviewing is the analysis of the round of interviews.

### 3.4 Analysing the round of interviews

Once convergent interviews were completed, analysis of the converged key issues for each round (group) of four interviews was conducted, ensuring all rural respondents were included in one separate round. Multiple-cross-case analysis was conducted to identify convergent patterns (Eisenhardt, 1989).

Qualitative data was imported and analysed using NVIVO and cross-triangulation amongst several data collection methods. The collected data was coded and categorised, including sorting data and initial coding of interviews. The importance of coding reliability has been highlighted in the literature as a focal point of thematic analysis (Clarke and Braun, 2017). Next, this coded data was analysed using inductive content and rigorous high-quality thematic analysis by enabling patterns, development of nodes, categories, subcategories and themes to emerge.

This *“method for identifying, analysing, and interpreting patterns of meaning (‘themes’) within qualitative data”* (Clarke and Braun, 2017, p. 297) provided systemic knowledge and insights on the CQI engagement of ED physicians and their perceived CQI culture. This approach enabled the comparison of themes and concepts to integrate data into one well-developed descriptive, theoretical framework. This computer-assisted qualitative data analysis software added value in developing a body of clinical knowledge in areas that require an in-depth understanding of multifaceted human interaction, such as ED CQI and CP.

The analysis of the round interviews included identifying common issues characterised in that round. For example, when the issue was identified by only one person in the round or raised in any other round/s just once, the issue was recorded but not considered a crucial subject matter to be pursued further in this study. Subject matter was considered important where two or more ED physicians in two or more rounds raised identical issues. Probing questions were also developed for key convergent issues, which allowed a deeper understanding of emergent themes. The probes were used to take advantage of opportunities, such as when respondents appeared to agree on key issues, but it was not clear whether the agreement was spontaneous.

### 3.5 Emerging themes

**Figure 1:**
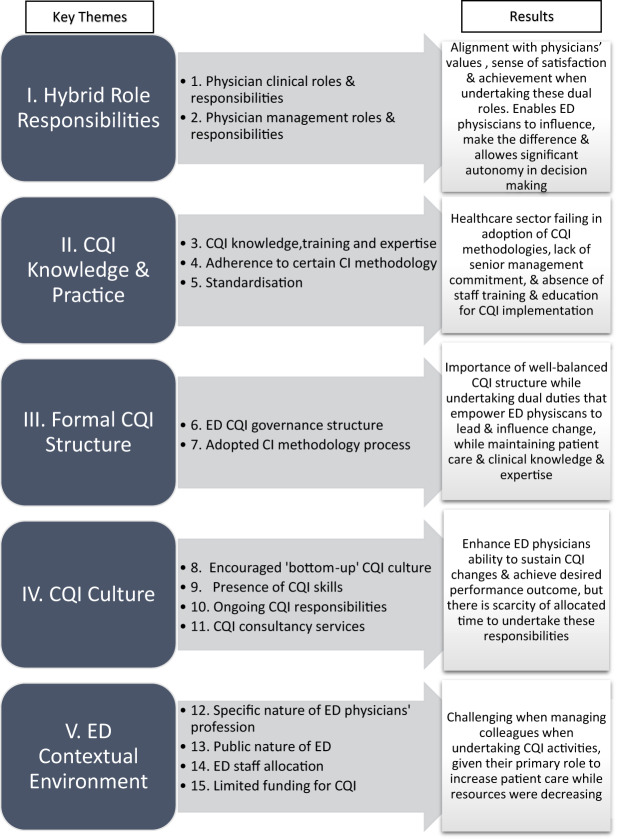
Key themes causing “tensions” when affecting perceived ED performance and study outcomes. Source: Authors’ own work

The five key areas and fifteen recurring themes emerged from the convergent interviews, including “Hybrid” role responsibilities, CQI knowledge and practice, CQI formal structure, CQI culture and ED contextual environment, as illustrated in Figure 1. The five key concepts affecting physician’s tensions are discussed in the following section.

#### 3.5.1 “*Hybrid*” roles and responsibilities

Physicians are often described as *“willing hybrids”* in leadership roles due to their clinical expertise and the hospital’s need for leadership in EDs (McGivern *et al.*, 2015). The work of *“willing hybrids*” has laid the foundation for *“changing and disrupting”* traditional medical professionalism and seek doctors managing EDs as well as retaining their clinical commitment for maintaining their peers’ validation of *“professionalism”* (McGivern *et al.*, 2015). The literature indicates that the concept of *“doctors in the lead”* is an effective strategy for monitoring and enhancing the quality of healthcare services delivered by medical professionals (Witman *et al.*, 2011, p. 491). According to the comment from one respondent from the convergent interview, the main advantages of physicians undertaking dual responsibilities are to allow physicians to be “*medically connected with patients on the one hand, but on the other hand gives some significant autonomy of decision making and control of their work, where the boundaries are very blurred.”*

The research also revealed that ED physicians lack CQI skills due to their well-established clinical “professional” culture underpinned by resistance to change to protect their professional values and knowledge (Mannion and Davies, 2018; Currie and Kerrin, 2004). As a result, long-term-employed ED staff see change as a threat. The interviewees highlighted that ED physicians are stuck in their old ways of doing things and resist implementing procedures as part of the CQI process. They dislike being challenged, especially by non-medical staff, in terms of their knowledge on management matters.

#### 3.5.2 CQI knowledge and practice

Our results revealed that there was no current expectation for ED physicians to lead and undertake ongoing CQI within their departments. However, where there was formal training and knowledge of CQI, there was departmental encouragement of the CI methodology, based on the Lean framework and the Plan Do Check Act (PDCA) practice (Lameijer *et al.*, 2023).

ED clinical staff expressed their view regarding the resistance to the CQI process. They felt that they had seen these types of projects before, and nothing has changed, stating that *“We did Lean but it did not work. This is due to an absence of understanding of Lean methodology and associating it with project work*.” Thus, there has been a challenge to get people motivated and actively involved in CQI activities within theirs EDs.

While the CQI process is straight forward to understand, its implication, to translate it into clinical care or to get clinicians engaged, was found to be particularly challenging: “*It is like bashing your head against a brick wall. The improved processes are difficult to implement. They are poorly adopted by people, and despite you do email, you have meetings, you hold seminars, you do face-to-face, you spend much time and effort trying to change things, but it is exceedingly difficult to implement them. This is where you need everybody involved in quality work, so they understand and support it.”*

The literature has emphasised the significance of standardisation of instructions, procedures and protocols, as important tools and the first step towards ongoing organisational improvements (Migita *et al.*, 2018). However, the current ED environment lacks standardisation according to 63% (*n* = 10) of the respondents. The 25% (*n* = 4) of EDs that had limited systems of standardisation found them complex and difficult to apply. The major reason for this was perceived to be the current ED physicians’ autonomous and dynamic nature of their clinical tasks “*SOPs are written outside our departments and therefore irrelevant and not applicable to our work and [so] no one is using them.”*

#### 3.5.3 CQI formal structure

Eighty-eight per cent (*n* = 14) of the respondents highlighted that there was no formal governance structure for encouraging and supporting CQI within their departments from the “bottom up”. Respondents revealed that there was an informal discussion due to ad hoc suggestions and activities. Even where CQI structure has been established (25% or *n* = 4), respondents emphasised this structure has not been fully operational, functional, and/or integrated, or allocated resources have been few and lack expertise. Just two participants have demonstrated a fully functional CQI structure that produces continuity and ongoing CQI culture. Notably, they highlighted that “*when the head of the department is unsupportive, it is very difficult or impossible to put appropriate CQI structure and culture in place.”*

#### 3.5.4 CQI culture

Respondents highlighted that clinicians may not be the best people to implement CQI due to their specialised CP focus. However, they pointed out that if clinicians are not involved, they are unlikely to be committed to creating a CQI culture. As one respondent noted, “*encouraging of bottom–up CI is challenging, … because of the position people occupy … and the hierarchies in which we work. The stakeholders must be involved, whether they are willing or unwilling*.” Consequently, there is the need for ED physicians to adopt a strategic approach. Thus, where CQI skills are lacking and CI concepts are not well understood by ED personnel, there is an absence of CQI culture.

Furthermore, participants revealed that CQI practices in rural EDs are rarely implemented, funded or supported by executive managers, nor has there been support for undertaking additional CQI training where necessary. Physicians from rural areas revealed that their level of engagement in non-clinical activities is extremely limited, lacking CQI training, specialist knowledge and support, causing ineffective CQI implementation. Our qualitative research revealed the need to prioritise the development of a CQI culture built on employee education of CQI tools and methods that value and encourage CQI activities. This would refocus the approach from CQI “tools” to creating systems, processes and policies that enable successful CQI implementation.

#### 3.5.5 ED contextual environment

The literature suggests that the ED contextual awareness and CQI culture leads towards positive performance outcomes (Dickson *et al.*, 2009). The ED contextual environment sets EDs apart from other private and public organisations, which seems to be contributing towards the physician’s tensions and resistance of CQI culture. This involves the autonomous nature of the ED profession with a very strong patient care focus, as stated by one of the respondents “*Everything that we do should be about improving patient care and there should never be an ethical question that has to be answered*.” However, the physician’s autonomous nature makes it difficult for them to adopt new ways and to think about the bigger picture and how change could benefit patients.

The current state of under-resourced EDs means they focus on CP and fail to prioritise CQI initiatives, particularly in rural areas, as stated by one respondent: “ *… there is a disparity of resources mostly through staffing, that doesn’t allow rural departments to improve because no one has any time to improve as you’re delivering health care as best you can with incredibly limited resources*”. These challenges are related to small ED centres in rural areas that have the same requirements as larger ED centres in terms of the range of responsibilities they are undertaking.

The remainder of this paper is concerned with the discussion of the research findings with respect to the research question we articulated earlier in this paper.

## 4. Results and interpretation of the findings

### 4.1 Study results

Interview discussions endorsed the most issues identified during the literature review process, indicating their relevance to current ED operational settings. For example, the results recognised healthcare sector failing in adoption of CQI management methodologies, lack of genuine senior management commitment and an absence of staff training and education for CQI implementation *(*Dickson *et al.*, 2009; Leite *et al.*, 2019).

The semi-structured interview data revealed a significant appetite to embrace implementation of CQI in EDs. While previous research has highlighted the presence of constant internal conflicts (“tensions”) for physicians due to their conflicting demands for CP and CQI (Kippist and Fitzgerald, 2009), the interview data emphasised alignment with physicians’ values and sense of satisfaction and achievement when undertaking these dual roles. The physicians reported valuable insights from their involvement in CQI endeavours, which they believed enhanced their ability to sustain CQI changes and achieve desired performance outcomes. However, the allocated time to sustain these responsibilities has showed to be a challenge.

Most respondents expressed the importance of well-balanced structure while undertaking their managerial duties that empowered them to lead and influence change, while maintaining patient care and clinical knowledge and expertise. For example, one clinician highlighted: “*I like the mixture. …. from the point view of patient work, because I can make bigger changes by doing an admin role. On the flipside, when I am doing the admin stuff, which can appear to be a bit cold and inhuman, the clinical work gives me the reason I am doing it, and they complement each other.”* Furthermore, the other added*:* “*I have lots of experience in different departments and therefore, I want to translate the best bits from all of my expertise and make my working place better as otherwise, you’re just turning up and seeing patients and having no impact*.”

In addition, respondents emphasised that their current managerial involvement enables them to influence and provides freedom for them to make the difference through CQI and allowed significant autonomy in decision making. One ED physician added: “*It is important for my role that I do admin role, as it helps me to understand what is happening in the departments, to stay connected with the staff that are working. It is important to make clinical currency and improvements in ED.”* All involved participants reflected favourably towards their required responsibilities and managed the paradox comfortably.

Notably, the respondents found it challenging when managing their colleagues as well as were required to undertake CQI activities, given their primary role to increase patient care while resources were decreasing.

### 4.2 Interpretation of the findings

The results revealed considerable inconsistencies of understanding of CQI processes in EDs, particularly with respect to CQI structure, knowledge and capability of CQI concepts by ED staff. This finding aligns with Dickinson *et al.* (2021) who found that the clinicians’ level of engagement in organisational quality programs and their contribution to be less than optimal, including a fragmented approach, and focusing on tools rather than strategically addressing CQI application in healthcare (Volochtchuk and Leite, 2022).

The above findings indicate that gaining CQI training and knowledge are key to building a CQI culture. However, in practice, CQI is more complex to achieve, given the barriers that exist for physicians in EDs even attempting to engage in something as simple and fundamental as “self-care” (to take their break or not) due the unpredictability of EDs and the obvious prioritisation of patients care (O’Shea *et al.*, 2020). This study outcomes confirm that whilst a range of benefits can result from implementing CQI, its application difficulties and complexities may prevent EDs from realising the full potential of CQI to create a culture of CI. Based on this finding, it is reasonable to assume that ED physicians redirect their resources to do what are most comfortable with, that is, doing their clinical duties and completely focusing on their patients, which is perfectly fine. However, according to Migita *et al.* (2018, p. 1294), physicians fail to realise the necessity for *“acceptance and support from all involved, especially leaders who can obtain and direct resources to the processes that enable important change to occur and be sustained”.* The physicians we interviewed reported valuable insights from their involvement in CQI endeavours, which they believed enhanced their ability to sustain CQI changes and achieve desired performance outcomes.

This research revealed that when physicians are not carrying out their clinical ED duties, tend to undertake CQI activities. This separation and off duty allow physicians to “*make bigger ED changes*.” This finding implies that staff members with non-clinical time allocation are likely to provide better patient care and implement CQI practice. Therefore, building CQI training and knowledge in addition to physicians’ clinical time duties can lead to ED changes and support building of CQI culture. O’Shea *et al.* (2020, p. 316) found that physicians should *“walk away from the space to be able to get some clarity and re-focus, then continue with their work”.* Thus, non-clinical time allocation for physicians to undertake these duties is imperative.

Furthermore, Berghout *et al.’s* (2017) argued that medical leaders’ time allocation to perform both roles appropriately is challenging, given their ever-scarce time and limited availability. The authors argue that for medical leaders to work effectively, they must manage the paradox by balancing their time to perform both roles (Berghout *et al.*, 2017). However, the dilemma of the extent and appropriate balance to which physicians must master role requirements remains unclear (Berghout *et al.*, 2017).

The study findings confirm the significance of the connection and interdependence of three major CQI dimensions: formal CQI knowledge, expertise and the adoption of CI methodology, all encouraging building a *CQI culture*. The research findings highlight the ED contextual environment as a significant factor in shaping the establishment a CQI culture and is one of the major factors helping to manage the paradox faced by ED physicians. This encompasses the specific nature of ED physicians’ profession, the public-facing aspect of the ED, staff allocation and engagement, as well as funding considerations. Whilst there is scarcity of literature directly linking the contextual environment of the ED to performance outcomes, existing research provides compelling evidence that the tensions, both between and within the core organisation’s activities, play a crucial role in driving the change and ensures the successful implementation and continuity of CQI (Smith and Lewis, 2011).

Likewise, prior research has identified public service external context as one of the common visible barriers (such as the emergency bureaucratic style, slow pace of change in health care context, public servant tenured career) for CQI implementation (Leite *et al.*, 2019). The evidence suggests that the challenge lies in the inability to effectively build and balance the CQI culture with the immediate clinical requirements of the ED.

## 5. Implications of research findings

The following recommendations aim to offer practical guidance and insights to the professional realm, thereby offering a valuable bridge between research insights and potential application of the findings. The implication of our findings for EDs is for CQI training activities to be incorporated as part of their criteria for promotion. The study provides practical insights to supplement existing quality improvement approaches currently led by EDs, which can potentially overcome obstacles of communication and collaboration in large public hospital EDs (Swastuti, 2023).

Most clinicians referred to CQI as “project work” that has a beginning and end. Furthermore, achievement of CQI cultural change in the behaviour and mindset of employees and leaders was perceived by respondents as a major challenge (Fournier *et al.*, 2021). These intra-professional separations restrict the implementation of CQI and its long-term sustainability in healthcare settings (Lindsay *et al.*, 2020). However, it is significant that both qualitative findings and the literature support the alignment between CQI training, knowledge and CQI formal structure with the establishment of CQI culture (Dickinson *et al.*, 2021; Volochtchuk and Leite, 2022). Perhaps, CQI should be understood by physicians as a continuous organisational journey that requires “an iterative and longitudinal approach” application (Migita *et al.*, 2018, p. 1294).

Learning across EDs proved to be difficult or non-existent due to lack of engagement of change agents. Hence, the practice reflects on ED physicians taking on formal leadership roles to engage in formalised CQI and drive collaboration throughout departments, meeting both, organisational and medical staff objectives, potentially improving organisational performance and patient care (Berghout *et al.*, 2017; Imran *et al.*, 2021; Orlando, 2020; Witman *et al.*, 2011). The implication for ED physicians, based on this study results, is that a common vision, commitment and support by “top ED executives” to the CQI process and “walking the talk” are imperative for CQI success. The ED executives and staff dedication for collaborative engagement for building CQI capability is an organisational necessity for the purpose of survival, growth and capacity to achieve tangible outcomes within an ever-changing environment (Dickinson *et al.*, 2021; Swastuti, 2023).

The use of CQI as a tool to help senior management to show a “quick fix solution” or “band aid” approach, instead of a philosophy of appropriate “bottom-up” CQI culture based on merit, is likely to result in implementation failure. The implication for ED physicians is that the CQI process requires time for appropriate implementation of identified improvement changes, so they provide tangible sustainable results. Allocating more of non-clinical time for clinical staff to undertake appropriate CQI activities would require more effective and efficient staff allocation planning process for their clinical vs non-clinical time. While this allocation might cause challenges at the short time and increase the required budget for additional staff, in long run it is expected that the sustainable and well led CQI activities will contribute towards time savings due to ongoing CQI process activities.

Implementation of CQI programs by physicians in many EDs was conducted in an “ad hoc” fashion without any centralised, defined and integrated approach at organisational level, or driven by state/national healthcare bodies (Dickinson *et al.*, 2021). As a result, this imposed a strain on the trust between physicians and their staff to effectively plan and implement CQI. The implication here is that this approach can lead to broadening of *“self-thought CI”* attitude instead of a *“collaborative and supportive CQI”* mindset amongst Australian rural and metro EDs. Overall, achievement of higher quality of patient care requires a change to a *“sustainable quality organisation”* by comprehensive management system, incorporating CQI tools built into supportive CQI institutional culture, and led by committed, supportive leaders (Imran *et al.*, 2021; Orlando, 2020; Prado-Prado *et al.*, 2020).

The literature supports the notion of understanding barriers throughout the journey of CQI transformation (Volochtchuk and Leite, 2022). Thus, the findings of this study provide practical benefits that would inform EDs and equip the wider healthcare sector with a greater understanding of the complexities and tensions surrounding EDs when adopting and implementing quality improvement strategies.

Finally, as intended, this study has developed theoretical propositions by exploring the paradox experienced by ED physicians which arises between clinical and CQI practice and provided insights of how perceived ED performance outcomes are affected. This awareness should encourage the healthcare sectors to act in embedding and driving of improvement strategy based on provided recommendations and findings.

Following the above discussion, the study has significant implications for developing a theoretical base for CQI practice implementation in Australian EDs. This study provides an extension of CQI as a theoretical foundation in EDs. Several researchers (Dickinson *et al.*, 2021; Lindsay *et al.*, 2020; Orlando, 2020) support the notion that there is the absence of empirical work examining physicians’ involvement in CQI for building staff engagement and addressing access block challenges.

## 6. Conclusion

The research outcomes revealed “tension” in the current ED environment, due to difficulty in allocating time to ED staff to perform their “CQI management” responsibilities while maintaining the highest standard of “clinical” duties in their pursuit of better patient care.

The lack of non-clinical time to develop CQI skills emerged as the main contributor of tension between CQI and CP in Australian EDs. This supports the concept of “paradox” for doctors when performing their required hybrid duties as medical experts and as managers for undertaking CQI activities.

With respect to the research question, we conclude that the physicians interviewed tend to lack CQI knowledge, coupled with insufficient allocation of non-clinical time to develop CQI capabilities. The physicians’ perception of the paradox was due to the constant staff resource limitations. Consequently, the provision of more non-clinical time to ED staff to be involved with CQI activities would enable the creation of a CQI culture within their EDs. Based on our qualitative research findings, it is reasonable to conclude that by adopting CQI management methodologies is likely to minimise “tension” and provide long-term improved ED performance outcomes. The opportunity for ED physicians to gain a greater understanding of the complexities and tensions within EDs would reduce resistance to change, while providing improved clinical care to meet community expectations and to reduce costs.

## 7. Opportunities for further investigation

The current research has demonstrated the complexity of balancing physicians’ clinical responsibilities with their managerial roles, leading to identity crisis (Sartirana *et al*., 2019). Further research could focus on a longitudinal investigation of the “hybrid” role requirements, of clinical vs non-clinical time allocation for ED physicians. This may allow risk avoidance of falling behind the expected level of ED managerial duties to ensure CQI continuity.

Future research could consider the cost–benefit of allocating non-clinical time to ED physicians for undertaking CQI activities. This analysis could help to convince healthcare sponsors to provide funding support for formal CQI training, expertise and to create a CQI formal structure, to encourage encouraging building a sustainable organisational CQI culture.
